# Proteomic Study on the Reproductive Toxicity of *Tripterygium* Glycosides in Rats

**DOI:** 10.3389/fphar.2022.888968

**Published:** 2022-05-20

**Authors:** Yanlin Dai, Lihui Sun, Shanshan Han, Shanshan Xu, Long Wang, Ying Ding

**Affiliations:** ^1^ Pediatric Medical College, Henan University of Traditional Chinese Medicine, Zhengzhou, China; ^2^ Department of Pediatrics, The First Affiliated Hospital of Henan University of Traditional Chinese Medicine, Zhengzhou, China

**Keywords:** *Tripterygium* glycoside tablet, proteomics, reproductive toxicity, molecular mechanism, testis

## Abstract

*Tripterygium* glycoside tablet (TGT) is a common clinically used and effective non-steroidal immunosuppressant. However, its reproductive toxicity limits its application in pediatric immune diseases, warranting the study of the molecular mechanism behind its reproductive toxicity. In the present study, 4-week-old male Sprague Dawley (SD) rats were provided TGT through continuous gavage with a clinically equivalent dose of 12 mg/kg for 12 weeks. The reproductive toxicity of TGT was recorded, and its toxicity mechanism was verified through experimental validation and proteomics analyses. Our results demonstrated that TGT could significantly reduce the testosterone level in the serum as well as the concentration and survival rate of sperms. Pathological sections of the testis revealed that TGT could reduce spermatocytes at different levels and make the convoluted meridians vacuolated. Based on tandem mass tag (TMT)-labeled quantitative rats testicular tissue proteomics, 34 differential proteins were screened, involving protein digestion and absorption, PPAR signaling pathway, PI3K-Akt, and other pathways, among which PI3K-Akt plays an important role in the study of reproductive injury. Western blotting results revealed that TGT could significantly downregulate the Col1A1, Col1A2, p-PI3K, and p-Akt expressions and inhibit the expression of proteins related to the PI3K-Akt signaling pathway. In summary, the clinically equivalent dose of TGT induced reproductive toxicity of 4-week-old male SD rats, possibly in relation to the inhibition of the PI3K-Akt pathway expression.

## Introduction

*Tripterygium* glycoside tablet (TGT) is the most widely used non-steroidal immunosuppressant in a clinical setting, owing to its anti-inflammatory, anti-tumor, immunosuppressive, and other effects (Song et al., 2020). It is widely used in rheumatoid arthritis (Zhang et al., 2021), connective tissue disease-related interstitial lung disease (Li et al., 2021), IgA nephropathy (Wang et al., 2017), purpura nephritis (Wang et al., 2018), atopic dermatitis (Liu et al., 2019A), and several other autoimmune and inflammatory diseases. Our previous research has also confirmed that TGT could significantly reduce the urinary protein level of children with purpura nephritis (Ding et al., 2019). Hence, it is established that TGT has a definite clinical effect. However, the chemical composition of TGT is complicated (Brinker et al., 2007); for instance, the therapeutic window is narrow and the patients are prone to multiple organ toxicity, involving the liver, kidney, and reproduction system (Qu et al., 2015; Jing et al., 2017), which significantly limits the clinical application of TGT. Indeed, reproductive toxicity is the main reason for the controversial application of TGT in pediatric immune diseases. Several clinical studies have confirmed that TGT can reduce the number and density of male sperm and that its long-term use can reduce the testicular volume (Su et al., 1987; Qian et al., 1989). In addition, animal studies with tripterygium wilfordii extract have shown that it induces sperm deformation, and testicular pathological damage and reduces sperm count (Ma et al., 2015; Chen et al., 2020). A past study showed that the oral administration of 0.1 mg/kg triptonide for 8 weeks led to >95% of abnormal sperm generation in adult male cynomolgus monkeys, with serious damage to the forward motile sperm. The fertility test revealed that both the female monkeys that matched 1:1 were not pregnant (Chang et al., 2021). Thus, TGT can cause male reproductive damage and even infertility, making it particularly important to study its reproductive toxicity mechanism.

Proteomics identifies or quantifies proteins and post-translational modification in biological samples on a large scale through high-efficiency protein identification technology. Once they are processed, the essential information is extracted from a high-throughput database (Ruotolo, 2022). This step provides a basis for the exploration of toxicity and efficacy mechanism. Among these, tandem mass tag (TMT)-labeled proteomics based on liquid chromatography-tandem mass spectrometry (LC-MS/MS) can accurately identify and quantitatively compare peptide-specific amino acids (Liu et al., 2020), which is of great significance to determine the drug toxicity and efficacy mechanism (Zhao et al., 2021; Song et al., 2022). In the present study, TMT-labeled LC-MS/MS technology was applied to explore the possible mechanism of reproductive toxicity caused by TGT to establish a basis for the clinical application of TGT.

## Materials and Methods

### Chemicals and Reagents

TGTs were purchased from Jiangsu General Pharmaceutical Co., Ltd. (Z32021007); Follicle-stimulating hormone (FSH), Luteinizing hormone (LH), Testosterone (T), and Estradiol (E2) kits were purchased from Elabscience Biotechnology Co., Ltd. Anti-Collagen alpha-1 (I) chain (Col1A1), Anti-Collagen alpha-1 (II) chain (Col1A2), Anti-phosphorylated phosphatidylinositol-3-kinase (p-PI3K), Anti-Phosphorylated protein kinase B (p-Akt), Anti-phosphatidylinositol-3-kinase (PI3K), Anti-protein kinase B (Akt), Goat Anti-Rabbit secondary antibody, and Goat Anti-Mouse secondary antibody were purchased from the ImmunoWay Biotechnology Company (United States).

### Animals

Four-week-old male Sprague Dawley (SD) rats were purchased from the Beijing Vital River Laboratory Animal Technology Co., Ltd. [SCXK (Beijing) 2016-0011]. The rats were fed under light and dark circulation for 12 h, and the animal tests were approved by the Ethics Committee of Henan University of Traditional Chinese Medicine (DWLL202105053). We attempted our best to reduce animal suffering as well as the number of animals used in accordance with Animal Care standards.

A total of 14 SD male rats were equally and randomly categorized in the control group and the TGT group. The control group rats were administered with normal saline through gavage, while the TGT group rats were provided 12 mg/kg of TGT suspension (grind 42 mg of *Tripterygium* glycoside tablets into powder and add normal saline to 35 ml) according to the gavage volume of 1 ml/100g, once a day. The experimental rats were continuously gavaged for 12 weeks and their weights were recorded every week. After fasting for 12 h, the rats were anesthetized with sodium pentobarbital, and serum was collected from the abdominal aorta. The level of sex hormone was detected by using the ELISA kit. The left epididymis was collected for the evaluation of sperm density and viability. The left testicular tissue was fixed with 4% paraformaldehyde for HE staining to observe any pathological changes. The right testicular tissue was stored at −80°C freezer for proteomics and Western blotting analyses.

### Whole Proteome Profiling by TMT-LC-MS/MS

Each group selected three biological replicates for proteomics analyses. The samples to be tested were homogenized with lysate (1.5% SDS/100 mM Tris-Cl, pH = 8.0) and then centrifuged to collect the supernatant. The protein in the solution was precipitated *via* the acetone precipitation method, and the protein concentration was determined by using the Bradford method. After protein quantification, 50 μg of the samples were subjected to SDS-PAGE detection, and the protein bands were observed after Coomassie brilliant blue staining. Trypsin was added based on the enzyme to protein mass ratio of 1:50 and incubated at 37°C overnight for enzyme digestion and peptide quantification (OD280). The peptide samples (100 μg) were labeled with TMT Mass Tagging Kit and then mixed equivalently with High-pH Reversed-phase Fractionation Kit (Thermo Fisher Scientific, Waltham, United States). First, the columns were equilibrated with acetonitrile and 0.1% trifluoroacetic acid (TFA). Second, the mixed TMT-labeled peptides were desalinated through low-speed centrifugation. Finally, the column-bound peptides were gradient eluted by high-pH acetonitrile solution with increasing concentration in turn and combined into 15 fractions. After vacuum drying, each eluted peptide sample was lyophilized with 12 μl of 0.1% FA and then stored in a refrigerator at −80°C, ready for machine detection.

We use Orbitrap Exploris™ 480 mass spectrometry (Product code: 725533, Thermo Scientific, United States) equipped with EASY-nLC™ 1200 system (Product code: LC140, Proxeon Biosystems, Thermo Fisher Scientific, United States) that can provide effortless peak performance for nanoflow applications up to 1200 bar. After dissolving the labeled peptide sample, it was inhaled through the automatic sampler and then passed through the analysis column (2 μm, 120 Å, 75 μm × 250 mm). The analytical gradient was established by using mobile phase A (0.1% formic acid) and mobile phase B (0.1% formic acid, 90% ACN). The linear gradient was 0–50% buffer B for 50 min, 50–100% buffer B for 5 min, and hold in 100% buffer B for 5 min. The flow rate of the liquid phase was set to 300 nL/min. During mass spectrometry DDA mode analysis, each scanning cycle included one MS full scan (R = 60 K, AGC = standard, max IT = 25 ms, scan range = 350–1500 m/z) and several subsequent MS/MS scans (R = 15 K, AGC = standard, max IT = 22 ms). Turbo TMT mode was adopted, with the cycle time set to 2 s. The HCD collision energy was set to 36 eV. The isolation window of the parent ion was set to 1.2 Da. The dynamic elimination time of ion repeated collection was set to 35 s. LC–MS/MS was performed by Sanshu Biotechnology Co., Ltd. (ShangHai, China).

### Data Collection and Analysis

The mass spectrum data were retrieved by using the MaxQuant Andromeda algorithm (V1.6.6) software, and the database used for retrieval was the proteome reference database of rats in the UniProtDuring (https://www.uniprot.org/). Fold change (FC) > 1. 2 or FC < 0.83 and Student’s t-test *p* < 0.05 served as the threshold for screening the proteins with significant differences. FC > 1. 2 indicated upregulation, while FC < 0.83 indicated downregulation. During functional annotation and enrichment analysis, based on the same sequence, GO term, KEGG pathway, and the cluster of orthologous groups of proteins (COG) corresponding to differential proteins sequence was obtained through the Diamond program of eggNOG-mapper.

### Statistical Analysis

All data were analyzed using the Graph Pad Prism (GraphPad, United States). The statistical levels of the two groups were calculated by an independent sample t-test for normal distribution and a nonparametric test for non-normal distribution. *p* < 0.05 was considered to indicate statistical significance, and all data results were expressed as mean ± standard deviation.

## Results

### General Indexes

**FIGURE 1:**
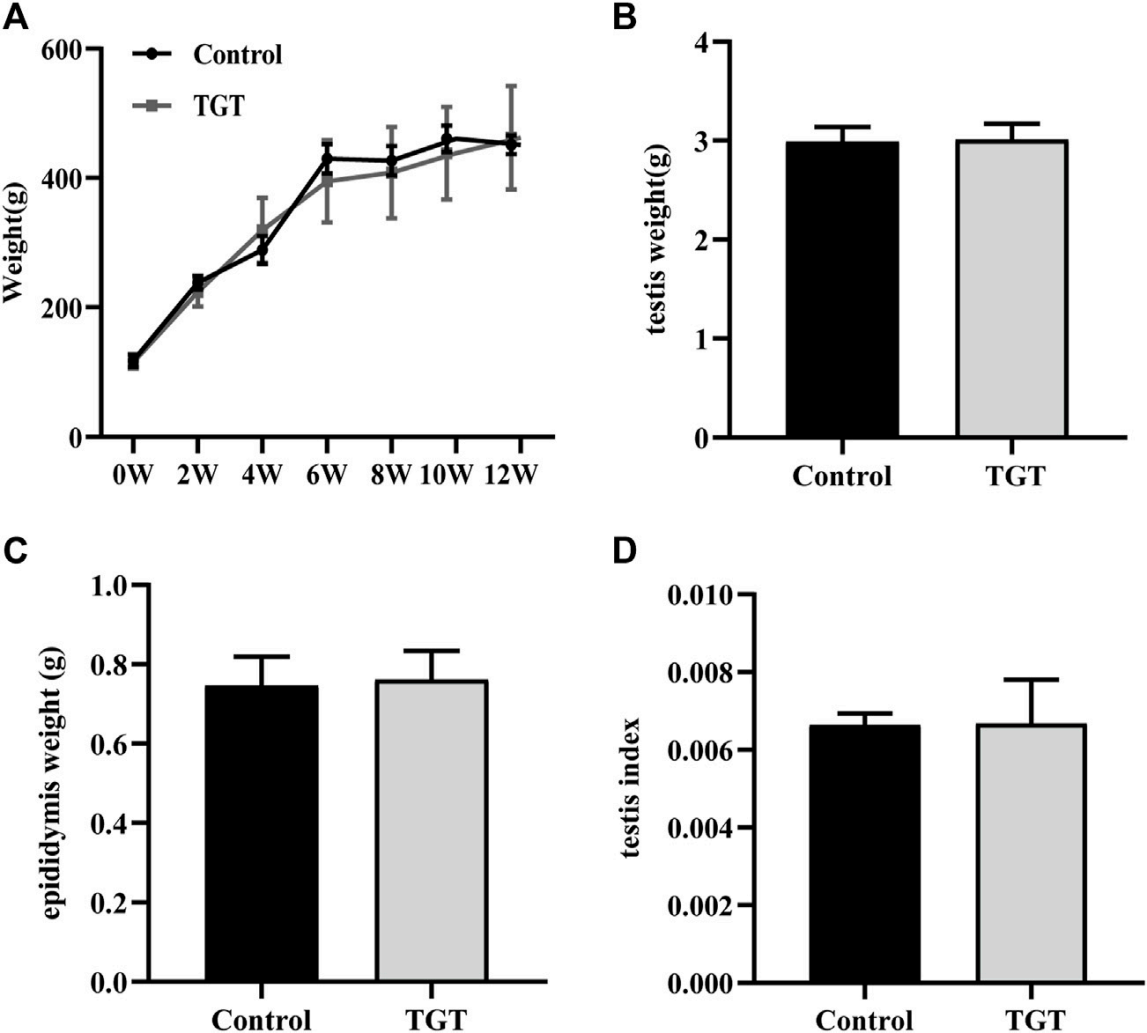
**(A)** Changes in the weights of the rats within 12 weeks. **(B)** The weights of the testis on week 12 of the experiment. **(C)** The weights of the epididymis on week 12 of the experiment. **(D)** The testis index on week 12 of the experiment. All values represent the means ± SD (*n* = 7).

In this study, the weight changes in the rats in the two groups were basically the same over 12 weeks (Figure 1A). In addition, compared with the control group, no significant decrease was noted in the testicular weight, epididymal weight, and testicular index in the TGT group rats (Figures 1B–D).

### Biochemical Indexes

**FIGURE 2:**
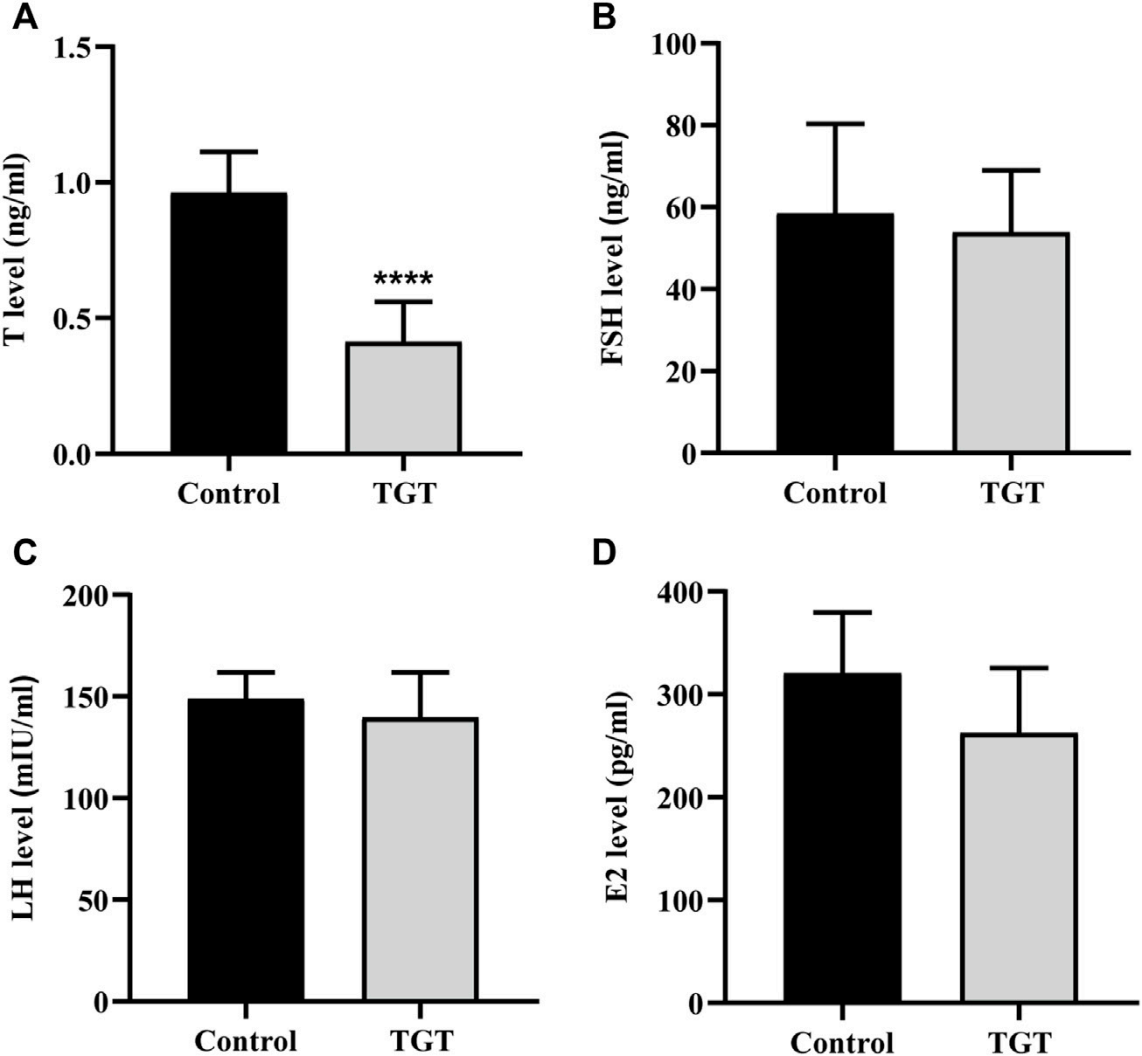
**(A)** Testosterone (T) level. **(B)** Follicle-stimulating hormone (FSH) level. **(C)** Luteinizing hormone (LH) level. **(D)** Estradiol (E2) level. All values represent the means ± SD (*n* = 7, compared with the Control group: ∗∗∗∗*p* < 0.0001).

As shown in Figure 2, clinically equivalent doses of TGT significantly decreased the testosterone level in the serum (Figure 2A), and the difference was statistically significant. The levels of serum FSH, LH, and E2 (Figures 2B–D) were not statistically significant between the two groups.

### Sperm Quality Index

**FIGURE 3:**
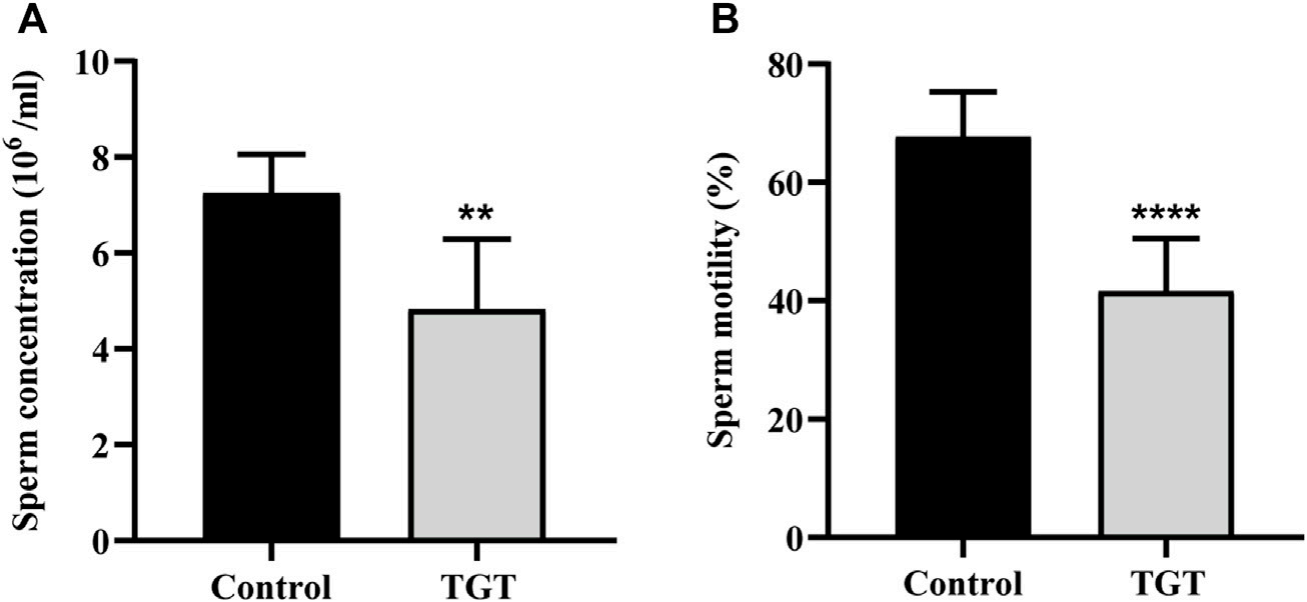
**(A)** The sperm concentration of the rats on week 12. **(B)** The sperm motility of the rats on week 12. All values represent the means ± SD (*n* = 7, compared with the control group: ∗∗*p* < 0.01 ∗∗∗∗*p* < 0.0001).

As shown in Figure 3, the TGT group showed significantly reduced density (Figure 3A) and activity (Figure 3B) of sperms in the epididymis, and the difference was statistically significant.

### Pathological Indexes

**FIGURE 4:**
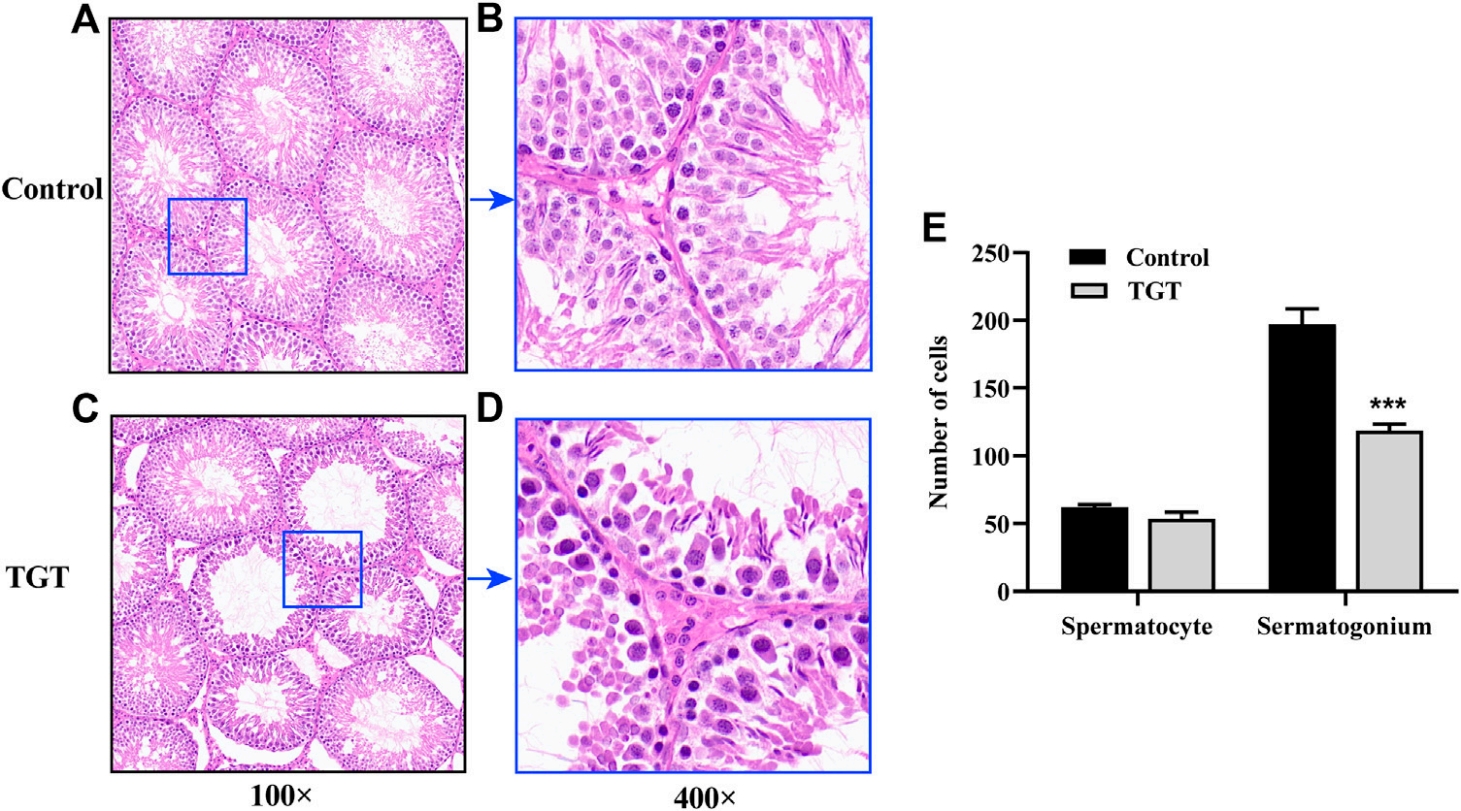
**(A)** Testicular pathology in the control group (×100). **(B)** Testicular pathology in the control group (×400). **(C)** Testicular pathology in the TGT group (×100). **(D)** Testicular pathology in the TGT group (×400). **(E)** Changes in the number of spermatogenic cells in the two groups. All values represent the means ± SD (*n* = 3, compared with the control group: ∗∗∗*p* < 0.001).

**FIGURE 5:**
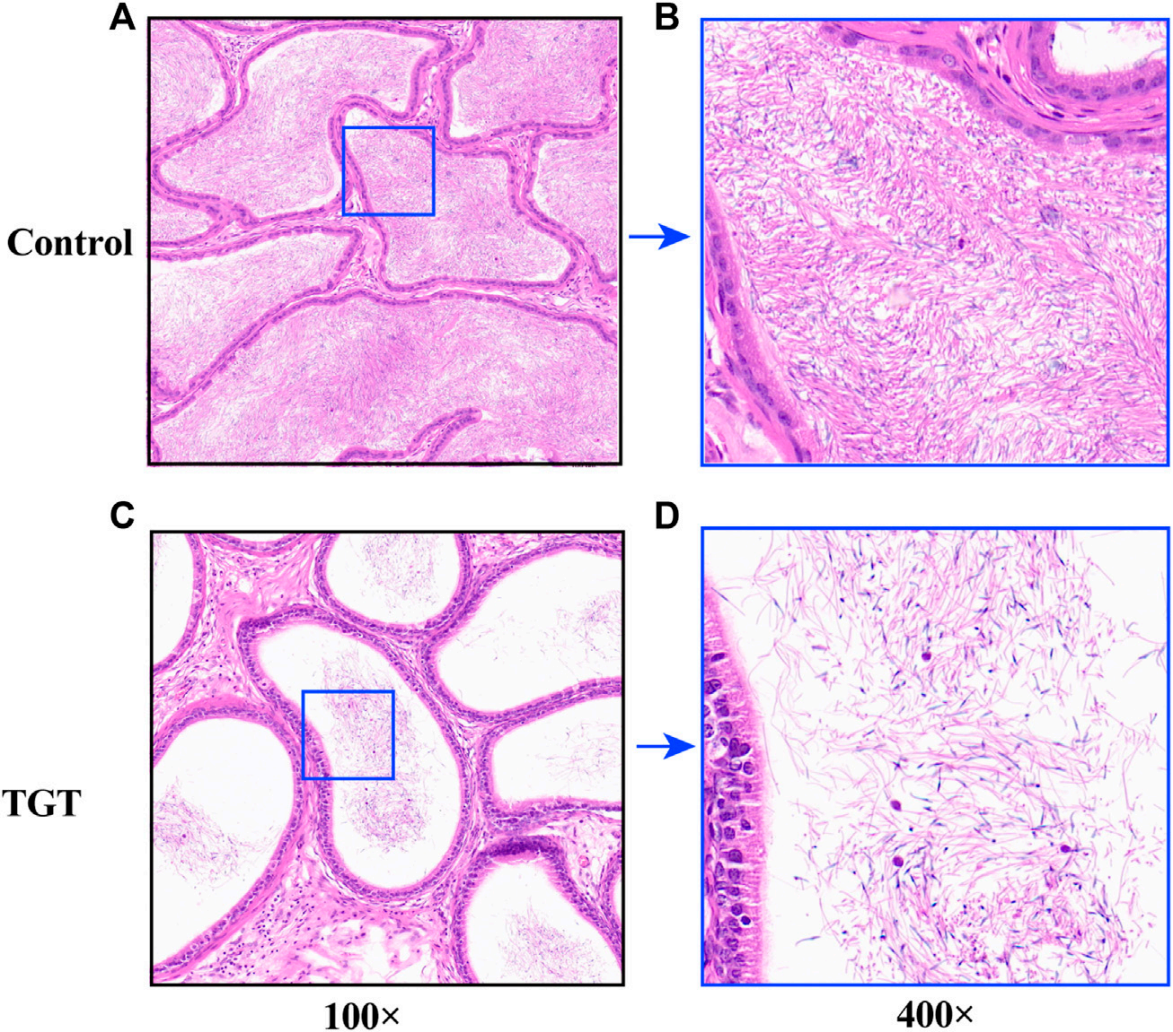
**(A)** Epididymis pathology in the control group (×100). **(B)** Epididymis pathology in the control group (×400). **(C)** Epididymis pathology in the TGT group (×100). **(D)** Testicular pathology in the TGT group (×400).

Figure 4 demonstrates testicular histopathology. The testicular seminiferous tubules of the control group rats were round or ovoid, with normal morphology. The wall of the seminiferous tubules was complete; the arrangement of the seminiferous cells was regular, compact, and clear; and mature sperm could be observed in the cavity (Figures 4A,B). However, the TGT group showed significant pathological damage, which was mainly characterized by atrophy of some of the seminiferous tubules, disordered arrangement of the spermatogenic cells, obvious reduction and the unclear number of layers, and reduction of the total number of spermatogenic cells at all levels in the seminiferous tubules (Figures 4C–E). Figure 5 illustrates the epididymal histopathology of the experimental rats. The diameter of the epididymal canal in the control group was large, and a considerable quantity of linear sperms could be detected in the lumen (Figure 5A), albeit the epididymal canal space of the TGT group had increased, with a significantly reduced size of linear sperms (Figure 5B).

### Proteomics Analysis

**FIGURE 6:**
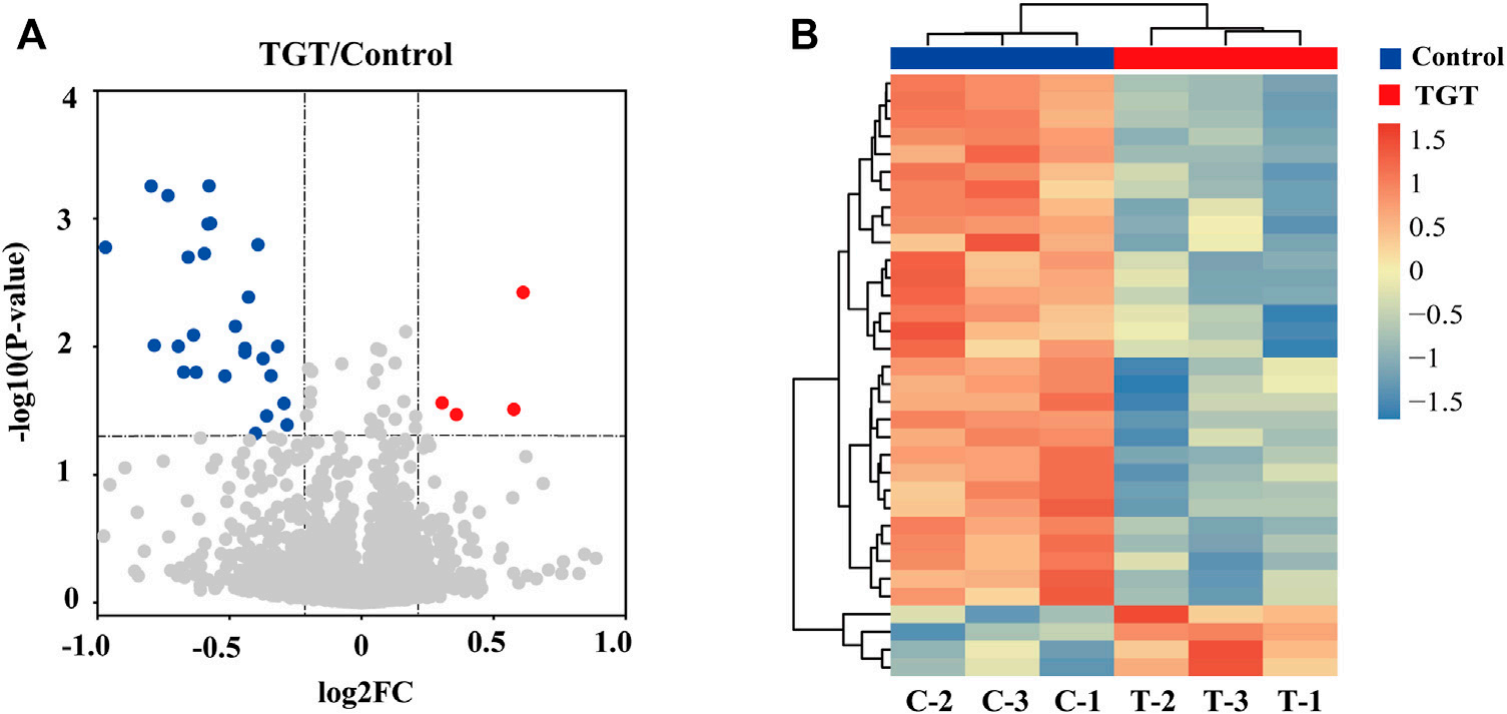
**(A)** Volcano plot for the TGT group versus control group in rats. The fold change >1.2 and *p* < 0.05 indicated differentially expressed proteins. Non-changed proteins are shown in gray color. Red color indicates upregulated proteins and blue color indicates downregulated proteins. **(B)** Heatmap plot of differentially expressed proteins. The color represents the relative expression of the protein in the sample; red represents a higher content in the sample, while blue represents a lower content.

**FIGURE 7:**
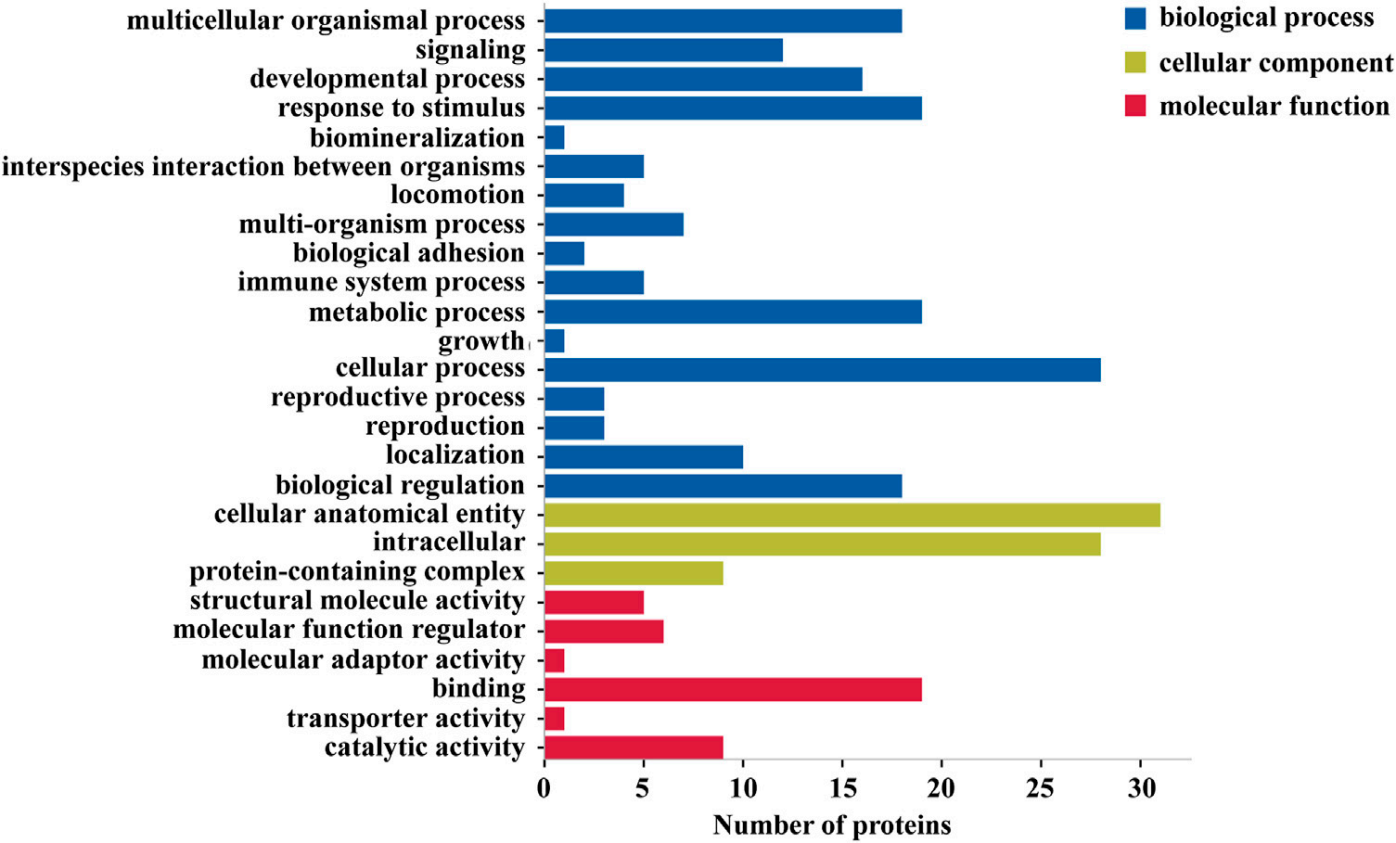
Functional enrichment of Gene Ontology (GO) annotation for differentially expressed proteins. Blue represents the biological processes, green represents cell components, and red represents molecular functions.

**FIGURE 8:**
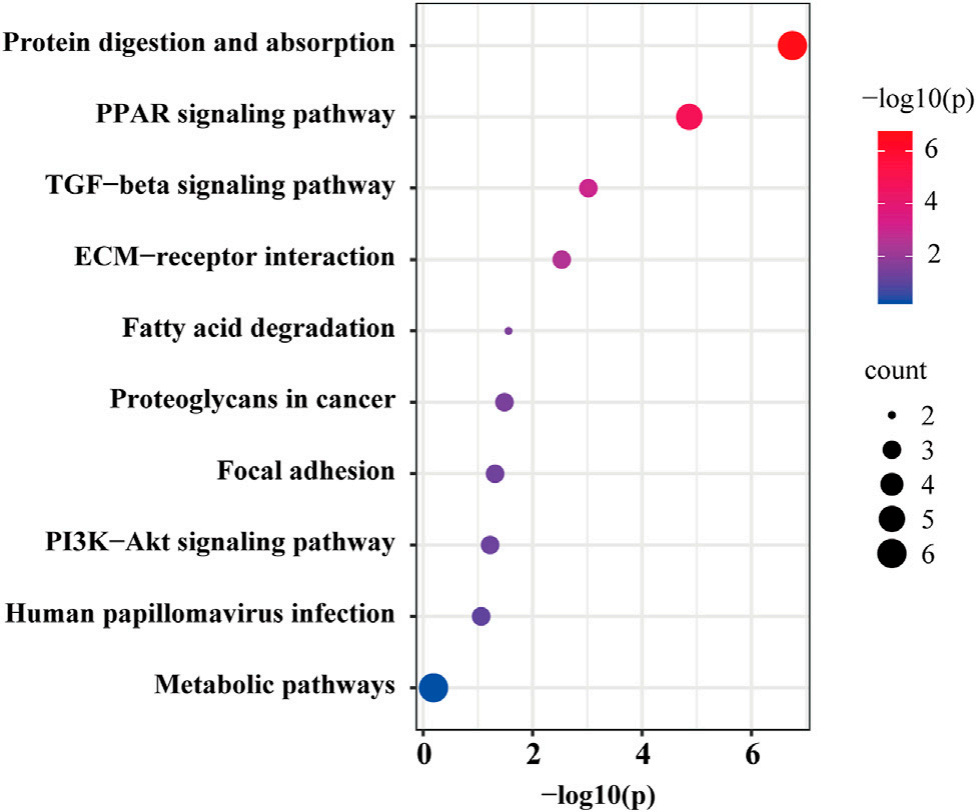
The KEGG pathway analysis between the TGT group and control group.

In quantitative proteomics analysis, differential proteins are usually screened according to the protein quantitative ratio and the *p* value of the statistical test. First, 7381 proteins were screened according to the fold change (FC) of the protein differential expression, with a fold ratio >1.2 (upregulation ratio >1.2 or downregulation ratio <0.83) and *p* < 0.05 (Zhu et al., 2022), and a total of 34 differentially expressed proteins were screened (Supplementary Table S1). When compared with the control group, the TGT group exhibited 4 and 30 upregulated and downregulated proteins, respectively. Figure 6A depicts a quantitative volcanic map of differentially expressed proteins. The screened differential proteins were further assessed by a heat map (Figure 6B). Figure 7 presents the GO comments of the identified differential proteins. As observed, the functions of these proteins were mainly catalysis, binding, transport activity, molecular function regulator, and participation in important biological processes, including multicellular interaction, signal transduction, stimulus-response, bio-adhesion, bioregulation, metabolism, development, and reproduction. The enrichment results of the KEGG pathway are shown in Figure 8. Significant changes were observed in terms of protein digestion and absorption, PPAR signaling pathway, TGF-β signaling pathway, ECM receptor interaction, fatty acid degradation, PI3K-Akt signaling pathway, and metabolic pathway.

### Regulation of PI3K-AKT Signaling Pathway

**FIGURE 9:**
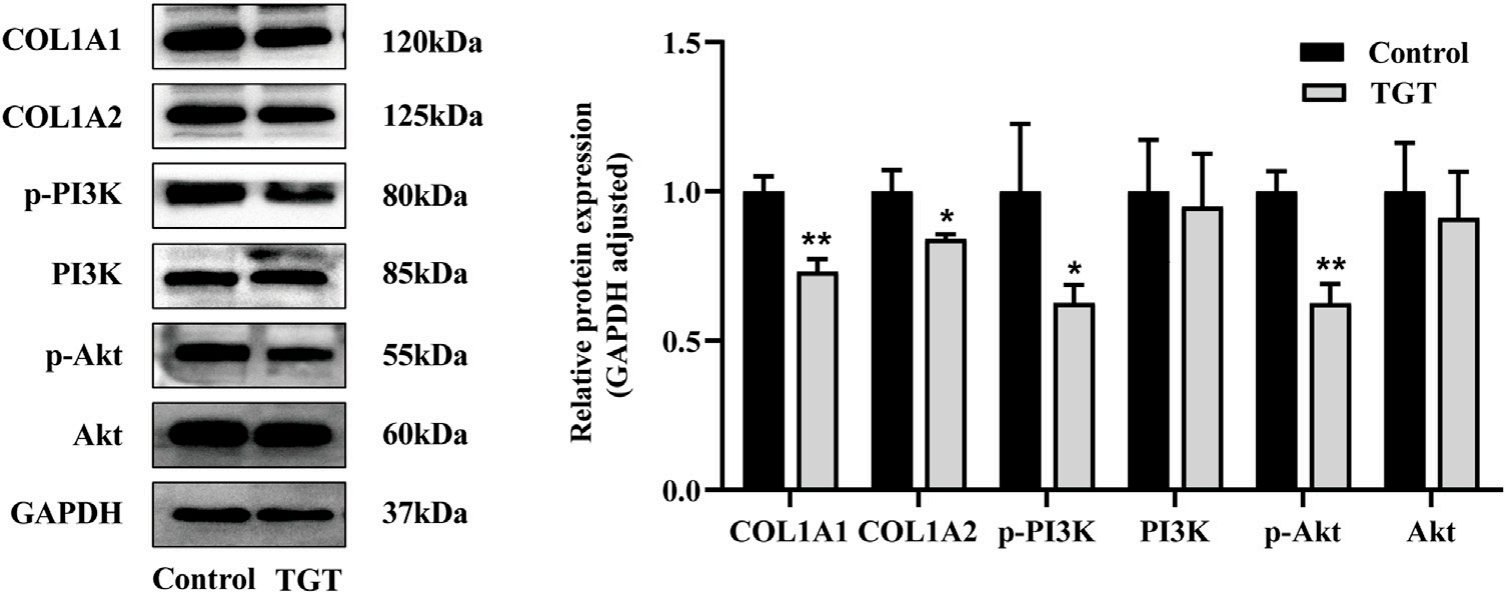
Western blotting of differentially expressed proteins, including the protein bands and relative abundance. All values represent the means ± SD (*n* = 3, compared with the control group: ∗*p* < 0.05 ∗∗*p* < 0.01).

Proteomics analysis revealed that TGT may produce reproductive toxicity by affecting protein digestion and absorption, PPAR signaling pathway, PI3K-Akt signaling pathway, and other pathways. The role of the PI3K-Akt signaling pathway in the reproductive process has been reported, with differential proteins Col1A1, Col1A2 (Supplementary Table S2), and major proteins of the PI3K-Akt signaling pathway such as p-PI3K, p-Akt, PI3K, and Akt employed for Western blotting. Our results demonstrate that TGT could significantly downregulate the expressions of Col1a1, Col1a2, p-PI3K, and p-Akt (Figure 9). Therefore, TGT may cause reproductive damage by inhibiting the PI3K-Akt signaling pathway.

## Discussion

TGT is the most widely used and reliable immunosuppressive Chinese patent medicine. It has been listed as a recommended medication for the treatment of proteinuria in Evidence-based Guidelines for the Diagnosis and Treatment of Purpura Nephritis (Trial) 2009 (Subspecialty Group of Nephrology et al., 2009) and also in the Medication Guidelines for Treatment of Rheumatoid Arthritis by *Tripterygium* Glycosides/*Tripterygium* Wilfordii Tablets 2020 (Lin et al., 2021). Nevertheless, it has a narrow therapeutic window and large toxic and side-effects (Liu et al., 2019B), which makes its clinical application controversial. Maximizing the efficacy and reducing the toxicity of this drug is thus of great significance for its safe administration in a clinical setting. Therefore, in this study, we focused on the molecular mechanisms of reproductive toxicity, which is the most worrying toxicity of TGT. Clinical studies have revealed that the average age of the onset of autoimmune diseases, such as purpura nephritis (Gomez et al., 2020; Pillebout and Sunderkötter, 2021) and juvenile idiopathic arthritis (Sag et al., 2019; Martini et al., 2022), is mostly 6–10 years, A 4-week-old SD rat is equivalent to an 8-year-old child (https://www.taletn.com/rats/age/). Hence, 4-week-old rats were enrolled in this study.

The main clinical manifestations of TGT toxicity to the male reproductive system are mainly manifested by the decrease in the testosterone level, oligospermia, azoospermia, the decrease in sperm activity, sexual desire, and reproductive function (Qian, 1987). Moreover, it has been demonstrated that TGT can significantly reduce the testosterone level, causing significant changes in the testicular pathology, including atrophy and distortion of the seminiferous tubules and a significant increase in the distance between adjacent tubules. Intraluminal observation has revealed that the densities of spermatocytes and sperms are greatly reduced (Jing et al., 2017). Similarly, our study found that the clinically equivalent dose of TGT exhibited significantly reduced testosterone level in the serum, which in turn decreased the sperm activity and density, albeit the decrease was smaller than that of high-dose TGT in the past studies, indicating that the reproductive toxicity of TGT is positively correlated with the dose of TGT. Thus, the pathology of the TGT group supports that TGT can cause testicular pathological damage and reduce the number of spermatogenic cells, which conforms with previous reports.

Proteomics analysis of the testis tissue samples of TGT-gavaged rats revealed that when compared with that in the blank control group, 34 proteins were significantly altered. Bioinformatics analysis revealed that these differential proteins were mainly enriched in protein digestion and absorption, the PPAR signaling pathway, and the PI3K-Akt signaling pathway. There are only a few studies on protein digestion and absorption pathway in germ cell injury (Guo et al., 2017). Proteomics has suggested that it may be involved in sperm production and differentiation. However, its specific role in male reproductive toxicity remains to be experimentally verified. The PPAR signaling pathway includes three important members: PPARα, PPARβ/δ, and PPARγ, all of which are expressed in rat testis (Braissant et al., 1996). Past studies have confirmed that the activation of PPARβ/δ prevents the expression of extracellular regulated kinase 1/2 phosphorylation and protects the testis from ischemia and reperfusion injury (Minutoli et al., 2009). In addition, the impairment of spermatogenesis may be caused by abnormal lipid and energy metabolism in testis *via* the downregulation of PPARs mediated by *Triptolide* (Ma et al., 2015). The involvement of the PI3K-Akt signaling pathway has been confirmed in multiple associations of male reproduction, including the regulation of the hypothalamic-pituitary-gonadal axis, the proliferation and differentiation of spermatocytes, and the regulation of sperm autophagy and testicular endocrine function during sperm development (Deng et al., 2021). Typically, the regulatory subunit gets activated after PI3K activation. Under the action of a catalytic subunit, the downstream effector molecule Akt gets activated, followed by the activation of the downstream target gene to promote the differentiation and proliferation of spermatogonial stem cells and the occurrence of sperms (He et al., 2009) (Supplementary Figure S1). In addition, PI3K-Akt is an important pathway that mediates autophagy and apoptosis. The inhibition of the PI3K-Akt signaling pathway induced excessive downstream autophagy and increased apoptosis, thereby increasing spermatogenic cell death and inducing damage to the male reproductive system (Huang et al., 2016; Zhang et al., 2017). On the other hand, TGT was found to be closely related to germ cell autophagy and apoptosis (Xi et al., 2017). Accordingly, the proteins enriched in the PI3K-Akt signaling pathway and the main pathway proteins of p-PI3K and p-Akt were verified. When compared with the control group, TGT downregulated the expression of Col1A1, Col1A2, PI3K, and Akt, which is consistent with the results of proteomics analyses.

In summary, TGT could inhibit the expression of proteins related to the PI3K-Akt signaling pathway, thereby reducing the quantity and activity of sperms and thus reproductive dysfunction.

## Conclusion

Our study results demonstrated that TGT could significantly reduce the testosterone level in the serum and concentration as well as the survival rate of sperms. The pathological sections of the testis revealed that TGT could reduce the number of spermatocytes at different levels, making the convoluted meridians vacuolated. Proteomics analysis was performed on TGT-gavaged rats’ testis tissue samples. When compared with the control group, TGT changed the expression of the related proteins. Further experiments demonstrated that TGT inhibited the PI3K-Akt pathway expression, exerting an anti-reproductive effect through the downregulation of Col1A1, Col1A2, p-PI3K, and p-Akt expression. The results of this study provide a reference for the reproductive toxicity mechanism and toxicity attenuation research of TGT.

## Data Availability

The datasets presented in this study can be found in the Supplementary Material and online repository iProX with accession ID IPX0004150001 (http://www.iprox.org).
