# Supplementary figures and images for: Proteomic Study on the Reproductive Toxicity of Tripterygium Glycosides in Rats
Source: Front Pharmacol. 2022 May 20;13:888968. doi: 10.3389/fphar.2022.888968 (PMC9163711; doi:10.3389/fphar.2022.888968)

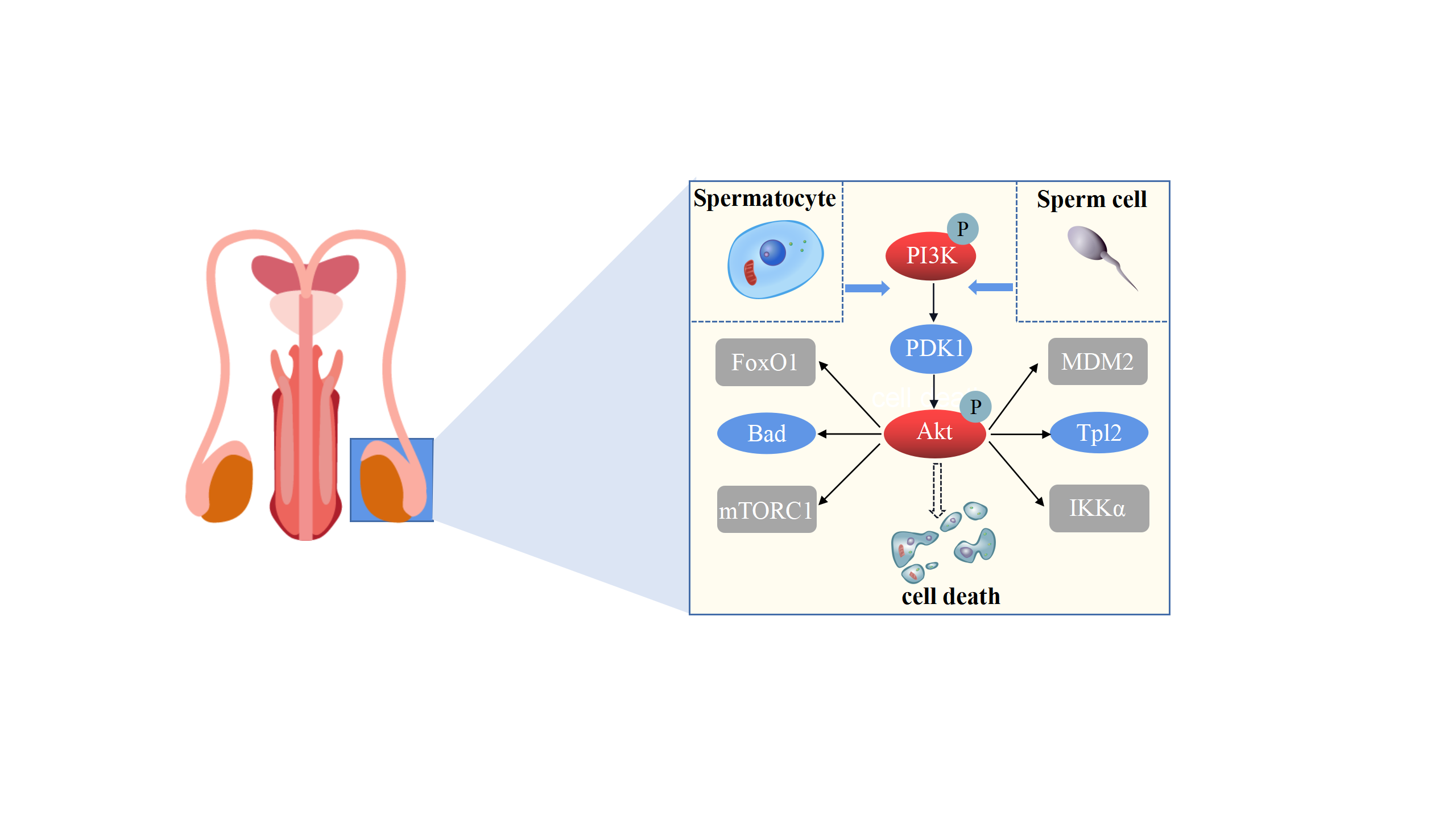

Supplement: Supplementary file 2 [file Image1.TIF]
